# Evaluation of Light-Emitting Diodes’ Effects on the Expression Level of P53 and EGFR in the Gingival Tissues of Albino Rats

**DOI:** 10.3390/medicina55090605

**Published:** 2019-09-18

**Authors:** Azhar Ghanim Ahmed, Alaa Hani Raziq

**Affiliations:** 1 Basic Sciences Department College of Dentistry, University of Sulaimani, Sulaimani 46001, Iraq; 2 College of Medicine, Duhok University, Duhok 42001, Iraq; 3 Pathology Department, College of Medicine, Duhok University, Duhok 42001, Iraq; ala_hani@yahoo.com

**Keywords:** dental curing lights, blue light hazard, P53, EGFR, qRT-PCR

## Abstract

*Background and objectives*: The light-curing unit is considered an essential piece of equipment in every dental office. This study was conducted to evaluate the effect of Light-Emitting Diodes (LEDs) by the light cure (LC) device on gingival tissues of albino rats histologically and by regarding the expression of P53 and epidermal growth factor receptor (EGFR). *Materials and methods*: Gingival tissues of the rats were exposed to LEDs for 30 s with an interval of 30 s for periods of 2 and 5 min and were examined after two and four weeks of light exposure. After the set time, histological sections were studied and the P53 and EGFR expressions were evaluated immunohistochemically and by molecular methods. *Results*: Mild hyperplasia and mild inflammatory response were detected in higher rates after two weeks of exposure when compared to 4 weeks postexposure. Whereas fibrosis was found at a higher rate after four weeks than that found after two weeks postexposure, parakeratosis was seen only in the group that was exposed for 5 min to LC and when biopsies were taken after 2 weeks. We found that the immunohistochemical expression of P53 was not changed. Similarly, the alteration of EGFR expression was statistically nonsignificant (*p* > 0.05) when compared to the control group. The data obtained from the qRT-PCR reaction was analyzed using the comparative CT (2^−ΔΔCT^) method. Statistically, there was no significant difference in the expression of EGER and P53 gene transcripts. *Conclusions*: LED causes no serious alteration in P53 and EGFR expression, and only trivial histopathological changes occurred, most of which recovered after a 4-week interval.

## 1. Introduction

Currently, various technologies use light in curing of dental composites, for instance, Halogen lamps, plasma arc lamps, lasers, and light-emitting diodes (LEDs). These technologies are different in their principles; light-emitting diode light-curing units (LCUs) are the most developed and commonly used devices by dentists [1,2]. The broader spectral output and high irradiation and power output of LED LCUs made the device the most popular; it can polymerize a wider range of resin by producing free radicals, initiating polymerization [3,4]. The dental light-curing unit is a fundamental device in dentistry; the device has been used in fields of restorative work, in luting cement in prosthodontics and adhesives in orthodontics, and in activating bleaching agents like hydrogen peroxide [5].

Light-curing devices not only affect the target tooth but also nearby tissues in the oral cavity. It has been reported that blue-light irradiation affects gingival tissues and significantly increases intracellular reactive oxygen species (ROS) levels and intracellular mitochondrial disorders [6]. Moreover, studies demonstrated cellular and subcellular changes when exposed to LED [7,8,9,10]. Visible light induced activation of the epidermal growth factor receptor (EGFR)-MAPK pathway and increased the levels of ROS and pro-inflammatory cytokines within the exposed tissues [11,12]. In vivo studies found that blue-light irradiation accelerates lipid peroxidation, which is an oxidative stress marker, by generating singlet oxygen (^1^O_2_) and ROS [13]. Deleterious effects such as cell death, apoptosis, carcinogenesis, and mutagenesis and mitochondria dysfunction could arise when cells are exposed to very high unregulated levels of ROS.

Epidermal growth factor receptor (EGFR) is a tyrosine kinase transmembrane receptor playing very important roles in biological cell functions such as cell division, differentiation, apoptosis, invasion, and contribution in the development and metastasis of cancer with developing resistance to chemotherapy [14,15,16]. EGFR is regarded as an early marker for the early detection of cancer in oral dysplasia [17].

P53 is encoded by a protein, TP53, and it is regarded as a tumour-suppressor gene found on the short arm (p) of chromosome 17 [18]. Its mutations are associated with one of the most popular oral carcinogenesis; mutation of P53 leads to the formation of the P53 protein that can be detected immunohistochemically [19]. Studies found that P53 plays an important role in developing oral squamous cell carcinoma (OSCC); P53 expression is linked to the epithelial dysplasia and squamous cell carcinoma SCC [20]. Furthermore, a significant correlation was reported between the expression of markers P53 and EGFR in leukoplakia and SCC [21].

This study aims to assess the expression of the P53 and EGFR markers by IHC using the qPCR technique and to study histopathological changes of the gingiva that are exposed to LEDs for different durations.

## 2. Materials and Methods

### 2.1. Materials

In the present study, a light-emitting diode (LED) device (JERRY® JR-CL17, Foshan, China) was used, which is a blue light-emitting source with a wavelength of 420–480 nm, a power output of 1000–1400 mW/cm^2^, and a frequency of 50–60 Hz. The diameter of the probe is 7 mm. The distance from the light source to gingiva of lower central incisors was 7 mm with light intensity 850–900 mW/cm^2^ that was measured by a photometer.

### 2.2. Animals

In this study, a total of 48 Albino Wistar male mature rats were aged 14–15 weeks and they were weighted 346.66 ± SD 32.67 g. Four animals were housed per cage and were maintained in a controlled environment at a temperature of 22–25 °C and under 12 h dark/12 h light cycles.

### 2.3. Experimental Design

The animals were selected randomly and divided into 6 groups of 8 animals each. The first group was considered as a control group 1 (not exposed to any manipulation), the 2nd and 3rd groups were exposed to light curing for 4 and 10 times (each exposure was for 30 s with an interval of 30 s) for 2 min and 5 min, respectively, and named light cure (LC) 2m 2w and LC5m 2w, respectively, and then, biopsies were taken after 2 weeks from the day of exposure. The 4th group was the control group 2 (not exposed to any manipulation), whereas the 5th and 6th groups were treated in the same manner as those of the 2nd and 3rd groups and named LC2m 4w and LC5m 4w, respectively, but the biopsies were obtained after 4 weeks from the day of exposure. Animals were anaesthetized before the exposure to LC using a mixture of ketamine and xylazine with specific doses.

### 2.4. Animal Sacrificing and Sample Collection

After the set time, rats were deeply anaesthetized and tissue biopsies were obtained from a labial gingival part of the lower central incisors. The taken samples were divided into two parts; one of them was put in RNA later solution, and it was saved in a deep freezer at −80 °C for molecular technique (RNA later™ Stabilization Solution, Invitrogen™, Carlsbad, CA, USA). The other parts of the tissue specimens were fixed in 10% neutral buffered formalin for 24 h for histopathological studies.

### 2.5. Microscopical Study

This procedure routinely was processed in the Histopathology Laboratory of Shorsh Hospital/Sulaymaniyah Governorate. Briefly, the gingival samples were embedded in paraffin blocks, and then, 3 sections were cut; one of them (4 μm thickness) was mounted on an ordinary slide for hematoxylin and eosin (H and E) staining for histopathological observation. On the other hand, the other two sections were mounted on positively charged slides for immunohistochemistry staining for the EGFR marker (pharmDx™ kit, Dako, Glostrup, Denmark IHC Detection System) and the P53 marker (Clone DO-7, Biogenex Life Sciences Limited, Fremont, CA, USA).

#### P53 and EGFR Scoring for IHC

Positivity for EGFR was considered when there was membranous and/or cytoplasmic brown staining, while P53 was nuclear brown staining. Negative and positive controls were included in this experiment. When <10% of the cells were stained for P53, it was considered as negative, and more than 10% was positive. Grading of P53 positivity was done according to the number of stained cells (1+: 10–30%, 2+: 30–50%, and 3+: >50%) [21,22]. EGFR was scored as 0, 1, 2, and 3 when 0% of cells were stained, <25% of positive cells were weakly stained, 26–50% of positive cells were moderately stained, and >50% of positive cells were strongly stained, respectively [23,24].

### 2.6. Molecular study

#### 2.6.1. RNA Extraction

Total RNA was isolated from gingiva tissue biopsies of albino Wistar rats using tissue total RNA Mini kit (Geneaid Company, New Taipei City, Taiwan) according to the manufacturer’s instructions. Tissue samples were efficiently homogenized in a microcentrifuge tube using a DNase and RNase-free Micropestle, before subsequent purification as described in the protocol. The RNA was eluted in DEPC-treated water and stored at −80°C. RNA quality and concentration were assessed using Biophotometer (Eppendorf, Hamburg, Germany) at wavelengths 260/280.

#### 2.6.2. Primer Design

A pair of primers for P53/Exp and EGFR/Exp was designed using an online software program: http://workbench.sdsc.edu. Glyceraldehyde 3-phosphate dehydrogenase (GAPDH) was used as an internal control gene (housekeeping gene) to normalize target genes P53 and EGFR. The primers used in this study are presented in Table 1.

Complementary DNA (cDNA) was constructed by Reverta-LRT reagents kit (K3-4-50-CE, Moscow, Russia). The annealing temperature of the oligonucleotides has been optimized by gradient PCR using a Bio-rad icycler iq5 PCR thermal cycler (Biorad, company, Philadelphia, PA, USA). The resulting PCR products were analyzed by 2% agarose gel electrophoresis stained with ethidium bromide. The gel was run at 100 volts for 45 min, and the cDNA fragments were visualized by UV-light. The optimal annealing temperatures were fixed as recorded in Table 1.

#### 2.6.3. One-Step qRT-PCR

This technique was used to construct cDNA and to amplify the target gene in a one-step RT real-time PCR protocol using SuPrimeScript qRT-PCR kit with SYBR green1 (GENETBIO Inc., Daejeon, Korea), which contains a complete system for fast, high yield and reliable single-tube real-time one-step RT-PCR.

The reaction mix was made in 20 µL, and reverse transcription reaction was performed using 50 ng/µL of the isolated total RNA, SuperScript RTas, and specific primers. Quantitative PCR reaction was done with a cDNA template all in one tube. Briefly, 4–5 µL of total RNA (50 ng/µL) was added to a 0.2-mL PCR tube; then, 1 µL of each forward and reverse primer (10 pmol/µL), 10 µL 2× reaction buffer, and 1 µL enzyme solution were added, and then, volume was completed to 20 µL by adding RNase-free water.

cDNA synthesis was done at 50 °C for 20 min, followed by amplification of the target genes as follow: one cycle of initial denaturation at 95 °C for 10 min, followed by 40 cycles of denaturation at 95 °C for 30 s, annealing at 55.6 °C for 30 s, and then extension at 72 °C for 30 s). Then, correctness of the resulting products was confirmed by a melting step using 55 °C to 95 °C with +0.5 °C increments for 30 s.

EGFR and P53 fold changes were calculated using the comparative cycle threshold (Ct) method. The average of the duplicate Ct values was used for analysis, and the target gene Ct values were normalized to those of the housekeeping gene encoding glyceraldehyde-3-phosphate dehydrogenase (GAPDH). Significance tests were calculated using the Wilcoxon signed-rank test of the duplicate 2^−ΔΔCt^ values for each gene in the control and Albino Wistar rat-exposed groups.

### 2.7. Ethical Approval 

This study was carried out with the approval of the Research Ethics Committee of Duhok Directorate General of Health in Kurdistan Region, Iraq under protocol number 4-29052028 on 29th May 2018.

### 2.8. Statistical Analysis 

Statistical analysis of the results was realized with IBM SPSS Statistics™ version 20.0 software (IBM Corporation, New York, NY, US), and an Excel sheet was used. The outcome of qRT-PCR included the comparison of fold means of each marker analyzed (EGFR and P53) in contralateral control samples using nonparametric statistics and Wilcoxon signed-rank test. On the other hand, the Chi-square test was used to evaluate EGFR expression in IHC.

## 3. Results

### 3.1. Histopathological Evaluation 

The changes within the gingival tissues of the control group and the LC-exposed groups were assessed on hematoxylin and eosin (H and E)-stained sections. Epithelial (ulcer, parakeratosis, hyperkeratosis, hyperplasia, and increase in clear cells) and subepithelial (inflammation and fibrosis) parameters were evaluated. As a result, we revealed that mild histological changes with no dysplasia were encountered in the exposed rats, and all the changes are summarized in Table 2.

### 3.2. P53 and EGFR Expression

**Figure 1:**
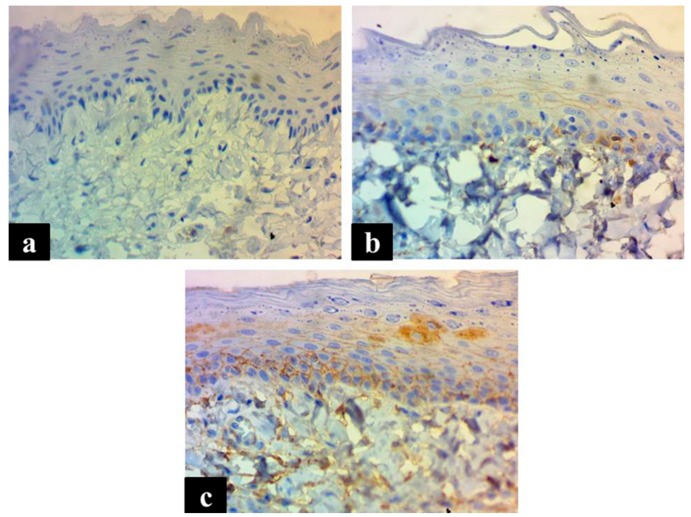
The photomicrographs show the expression of EGFR in both membranous and cytoplasm as an accumulation of a brown EGFR protein. EGFR expression scored as (**a**) score 0, no expression; (**b**) score 1+, <25% positive cells for weak staining; (**c**) and score 2+, 26–50% positive cells for moderate staining. Magnification ×400.

P53 was not expressed in any tissue sample. Regarding EGFR expression, the results are shown in Table 3 with the clear absence of score of 3. Figure 1 shows the expression of EGFR with scores of 0, 1, and 2. There was no statistically significant difference in the intensity and extent of EGFR expression between control and LC-exposed groups.

### 3.3. Effect of LED on the Expression of Both P53 and EGFR Genes

**Figure 2:**
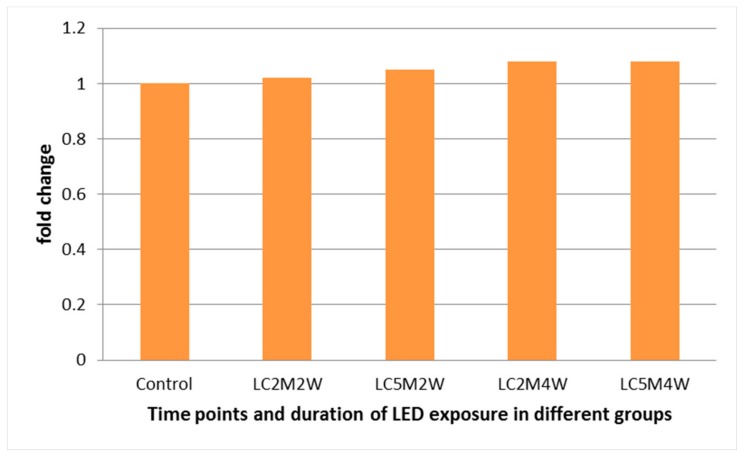
Fold change in the expression of P53 in gingiva tissues at the corresponding time points and duration. M: minutes, W: weeks, and LC: light cure.

**Figure 3:**
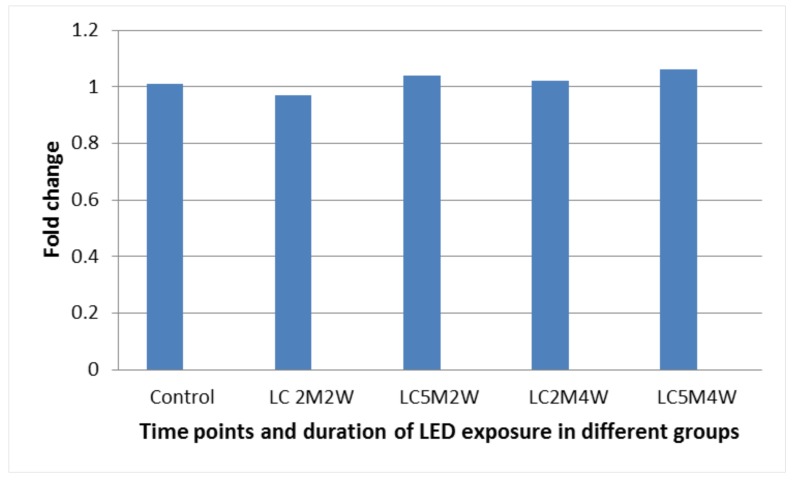
Fold change in the expression of EGFR in gingiva tissues at the corresponding time points and duration. M: minutes, W: Weeks, and LC: light cure.

The effect of LED was assessed by quantification of the relative expression of P53 and EGER gene transcripts in gingival tissue biopsies as a fold change (Figure 2 and Figure 3). Our results show that LED will not affect the expression of both P53 and EGER compared with the control group because there was no significant difference in fold changes of these two genes with the control group in gingival tissues after LED exposure. Gingiva taken from the LED exposure group, compared to the unexposed control group, at each sample time point, revealed no significant difference (*p* > 0.05) between the two groups for both genes, see Figure 2 and Figure 3.

## 4. Discussion

In general, few studies have focused on the effects of blue-light irradiation on gingival tissue. Therefore, this work aimed to study the effects of blue-light irradiation on gingival tissue using LED. To best of our knowledge, no previous effort has been done to explore gingival tissue changes after exposure to light-curing devices used in the dental field by IHC and the molecular technique.

Blue light has the shortest wavelength of all types of visible light (380–495 nm). Accordingly, blue photons have greater energy than photons with longer wavelengths and higher frequency. Blue light is sometimes referred to as high-energy visible light [25,26].

The current study revealed mild histological changes (hyperplasia, hyperkeratosis, parakeratosis, clear cell, and inflammation) within the gingival tissues; these findings are in line with previous studies [7,8,9,10]. Despite two different light-curing exposure times (2 min and 5 min) in this study, the data did not find any dysplasia or ulcers. These findings disagree with Reference [7], that used 2 min and in which was reported 10% and 4% basal layer atypia and ulceration, respectively.

Visible light from the curing unit gives thermal and photodynamic stimuli, resulting in an inflammatory response [27]. Therefore, in the present study, most of the pathological changes decrease in severity from 2-week groups to 4-week groups.

In the present study, no difference was found within hyperkeratosis between different exposure times and time points postexposure. These data do not match with that reported by Reference [9], who found a progressive decrease in epithelial thickness in a study using very little distance (2 cellulose strip thickness) between light cure source and gingival tissue in several biopsies taken from the 1st to the fifth day.

In this study, all cases of normal oral gingiva showed no P53 expression. This is following the studies done by References [20,28,29,30]. This could be due to the short half-life of P53 and lack of stabilization in normal mucosa [31]. Seventy-five percent of LC 2m 2w and LC 5m 2w showed hyperplasia; however, no significant difference was found in P53 expression compared with the control group. On the other hand, Reference [32] demonstrated that P53 was expressed in 36% cases of hyperplasia. The differences could be due to the severity of hyperplasia, where severe hyperplasia was reported by Reference [32].

Similar to P53, this study did not find significant scores of EGFR expression compared to the control group. A definite correlation was observed between P53 and EGFR. This may be explained by the fact that both P53 and EGFR are interlinked to each other at a molecular level and may augment each other in cases of dysplasia and carcinogenesis [21]. Wang et al [33] showed that the mutant P53 binds to promote a sustained EGFR-induced extracellular signal-regulated kinase 1/2 activation, thereby facilitating cell proliferation and tumorigenesis.

## 5. Conclusions

From the present study, we concluded that LED causes no serious alterations in P53 and EGFR expressions, and only trivial histopathological changes occur, with most of them recovering after a 4-week interval.

## Figures and Tables

**Table 1:** The list of primers for gene expression determined by qPCR.

Primer Name	Sequence 5′–3′ for Forward Primers 3′–5′ for Reverse Primers	Optimal Annealing Temperature	PCR Products Size	References
P53	F: GTCACGCTCCCCTGAAGAC	Present study	270 bp	55.6 °C
	R: CAGGAGCTGACACTTGGAGG			
EGFR	F: ATTAATCCCGGAGAGCCAGAG	Present study	157 bp	55.1 °C
	R:GTGAGCCTGTTACTTGTGCC			
GAPDH	F: AGTGCCAGCCTCGTCTCATA	Liang et al. 2018	248 bp	55.6 °C
	R: GATGGTGATGGGTTTCCCGT			

Abbreviations: EGFR: Epidermal growth factor receptor; GAPDH: Glyceraldehyde 3-phosphate dehydrogenase; bp: base pair.

**Table 2:** The effect of Light-Emitting Diode (LED) (light cure (LC) 2m and LC 5m) on histopathological parameters in Albino Wistar male rats at 2-week and 4-week time points postexposure compared to control groups.

Parameter %	Control Groups (1 and 2)	LC2m 2w	LC2m 4w	LC5m 2w	LC5m 4w
Ulcer	0	0	0	0	0
Increase in clear cells	0	62.5	25	75	25
Hyperkeratosis	0	25	25	25	25
Parakeratosis	0	0	0	62.5	0
Hyperplasia	0	75	37.5	75	37.5
Dysplasia	0	0	0	0	0
Inflammation	0	62.5	25	87.5	25
Fibrosis		0	37.5	37.5	50

M: minutes, W: Weeks, and LC: light cure.

**Table 3:** Scores of EGFR protein expression in the animal groups (Magnification ×400). M: minutes, W: weeks, and LC: light cure.

Group	Score 0	Score 1+	Score 2+	Score 3+
Control 1	2	5	1	
LC2m 2w	2	6		
LC5m 2w	1	7		
Control 2	3	5		
LC2m 4w		6	2	
LC5m 4w		6	2	
